# Profile of cancer patients requiring dental and oral-maxillofacial prostheses in a Brazilian subpopulation

**DOI:** 10.4317/jced.59112

**Published:** 2022-02-01

**Authors:** Cristiane L. Garcia, Ivan O. Gialain, Maria CPF. Volpato, Luiz ER. Volpato

**Affiliations:** 1 DDS, MSc, University of Cuiabá, Cuiabá, Brazil; 2 DDS, MSc, PhD, University of Cuiabá, Cuiabá, Brazil; 3 DDS, MSc, University of Cuiabá and Mato Grosso Cancer Hospital, Cuiabá, Brazil; 4 DDS, MSc, PhD, University of Cuiabá and Mato Grosso Cancer Hospital, Cuiabá, Brazil

## Abstract

**Background:**

The aim of the present study was to identify the profile of cancer patients in need of rehabilitation with dental and/or oral-maxillofacial prostheses and evaluate possible reasons for not concluding the rehabilitation process.

**Material and Methods:**

A retrospective observational study was conducted at the Dentistry Department of the Mato Grosso Cancer Hospital in the city of Cuiabá, Brazil, involving the analysis of the medical records of patients under care from April 2017 to November 2019.

**Results:**

The study population comprised 256 patients who met the inclusion criteria. A total of 30.90% of the patients were older adults, 65.6% were men, 70.3% had brown skin color, 27.3% were retired, 49.2% were married and 52% resided in municipalities in the state of Mato Grosso other than the capital. A total of 67.23% reported smoking and 53.9% reported alcohol consumption. The tumor was located in the head and neck region in 57.4%. The most frequent histological type was epidermoid carcinoma (55.1%). A total of 28.9% of cases were in disease stage IV. Most patients (60.2%) completed prosthetic rehabilitation, with a predominance of total prosthesis. The main reasons for not completing rehabilitation were the patient’s death and weakness.

**Conclusions:**

Patients who started treatment in more advanced stages of cancer had a greater chance of not completing the prosthetic rehabilitation. The non-completion of treatment was directly related to death and the state of weakness.

** Key words:**Cancer, dental prosthesis, epidemiology, maxillofacial prosthesis, oral rehabilitation.

## Introduction

Cancers of the head and neck region profoundly affect the quality of life of patients due to the effect on esthetics, serving as a constant reminder of the disease. These cancers are emotionally draining for patients and their families (1).

Indeed, the diagnosis of cancer exerts a negative impact on the patient’s life. Fear and suffering are common throughout the process, which begins with the diagnostic phase and is followed by therapy and survival (2,3). Most post-treatment patients whether surgical or not remain in follow-up for an average of 10 years (4).

Cancer treatment has side effects ranging from mild to severe (5). Large facial defects compromise vital functions such as breathing, chewing, speaking and swallowing, as well as esthetics. The prosthetic reconstruction of facial defects helps restore function and assists in the recovery (6).

In the treatment of tumors of the head and neck region, patients are also often submitted to surgeries that have a serious impact on quality of life, appearance and functional characteristics (7). Mutilations of the face can have important esthetic and functional impacts, causing morphofunctional and psychosocial changes that can lead to social isolation. Thus, it is imperative for healthcare providers to commit to the rehabilitation of such patients (8).

Prosthetic treatment is indicated for the recovery of lost oral functions and an improvement in physical appearance, enabling patients to participate in daily activities with greater confidence (9). The absence of teeth not replaced by a prosthesis also exerts a negative impact on the quality of life of cancer patients (10).

Given the need for dental care among cancer patients and the positive impact of rehabilitation on their quality of life, the aim of the present study was to outline the profile of cancer patients rehabilitated with dental and/or oral-maxillofacial prostheses and investigate reasons for the non-completion of rehabilitation process.

## Material and Methods

-Population 

Data collection involved the analysis of the medical records of patients treated at the Dentistry Department of the Cancer Hospital of Mato Grosso in the city of Cuiabá, MT, Brazil, from April 2017 to November 2019. This period was selected because the hospital currently uses a single medical record for each patient and the medical records are managed by an information system that was installed in April 2017.

A search for relevant cases was performed in the Care Management System for the determination of all patients with an indication for prosthetic rehabilitation treatment in the period. Patients of both sexes and of any age were included. Patients having undergone rehabilitation but with no confirmed diagnosis of cancer and those for whom it was not possible to collect data from the respective medical records were excluded.

-Data collection

Data were collected on age, sex, skin color (defined based on the recommendations of the *Instituto Brasileiro de Geografia e Estatistica* Brazilian Institute of Geography and Statistics]), place of residence (state capital or other municipality), occupation, marital status, smoking habit, drinking habit, family history of cancer.

Characteristics of the disease (tumor location, histological type, stage of the disease and treatment performed [surgery, radiotherapy and chemotherapy]), characteristics of prosthetic rehabilitation (type of maxillary prosthesis [total, partial, total obturator and partial obturator], mandibular prostheses [total or partial], and facial prostheses) and reason for not completing rehabilitation.

Patients who met the inclusion criteria but whose medical records did not present information on completing rehabilitation or the installation of the prosthesis were actively contacted by telephone and asked about the reason for not completing rehabilitation treatment. The answers (provided by the patient or family member) were placed into five distinct groups: patient died before completing rehabilitation treatment; patient interrupted treatment because of weakness; patient is still undergoing rehabilitation treatment; patient completed the rehabilitation elsewhere; and no defined response (cases for which telephone contact was unsuccessful).

-Data analysis

A single researcher collected and organized the data on an Excel spreadsheet. Descriptive statistical analysis was performed for the variables of interest. The data were expressed as absolute and relative frequencies. Multinominal logistic regression was performed to analyze possible associations between the independent variables and dependent variables (not completing prosthetic rehabilitation and reason for not completing rehabilitation), as described elsewhere (11). Statistical tests were performed using the SPSS program, version 22.0. Results with a *p-value* <0.05 were considered statistically significant.

## Results

Four hundred seventy hospital records were analyzed, 256 of which met the inclusion criteria. Exclusions were due to the non-confirmation of a diagnosis of cancer or the impossibility of collecting data from the medical record.

Age in the sample ranged from 30 to over 70 years. The highest percentage of patients (30.9%; n = 79) was between 61 and 70 years of age. The least frequent age group was 30 to 40 years of age (2.7%; n = 7). Male patients predominated (65.6%; n = 168) over female patients (34.4%; n = 88). The most prevalent skin color was brown (70.3%; n = 180). Almost half of the patients resided in the state capital (48%; n = 123) and slightly more than half resided in other municipalities of the state (52%; n = 133). Seventy patients (27.3%) were retired; 126 patients (49.2%) were married; 172 patients reported smoking (67.2%), 138 patients (53.9%) reported alcohol consumption and 130 (50.8%) had a family history of cancer (Table 1).

**Table 1:**
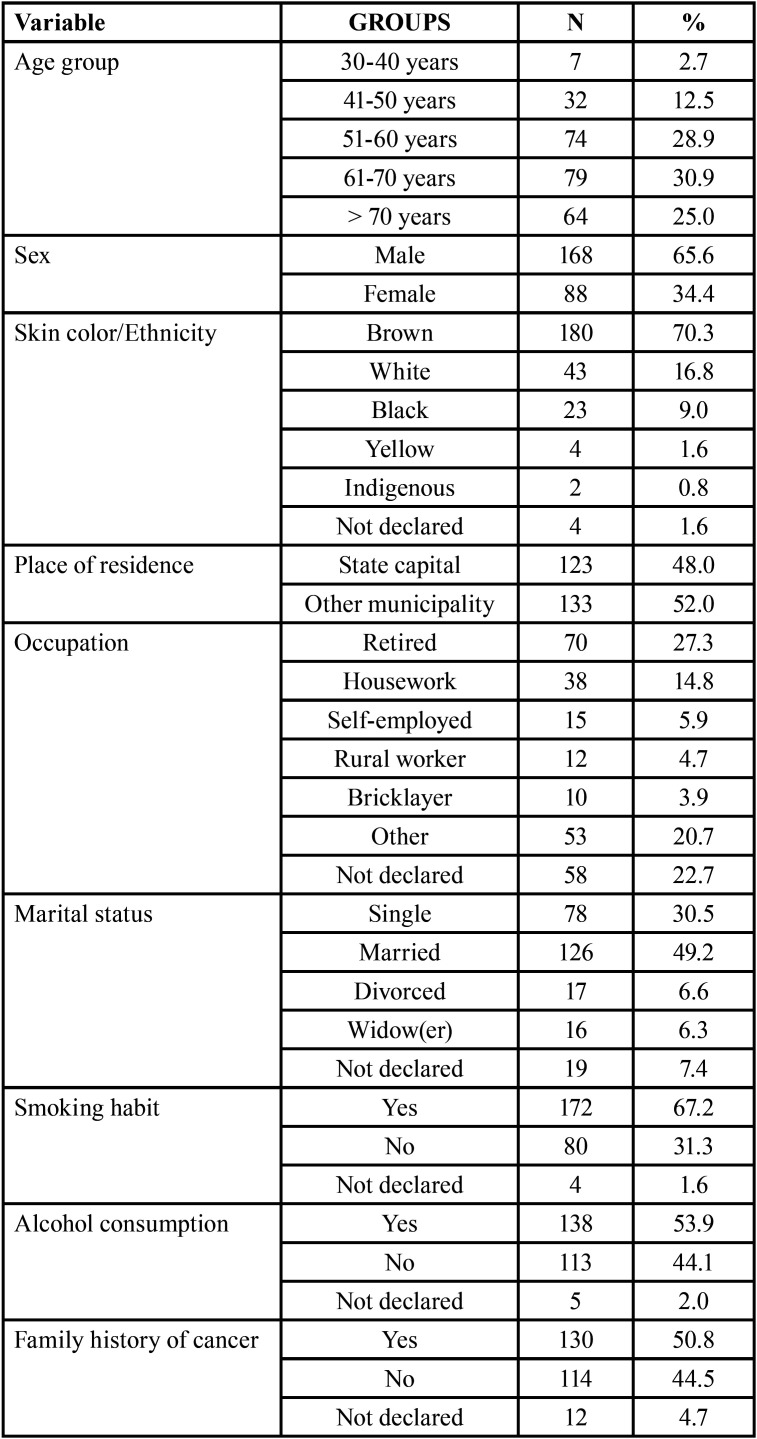
Distribution of patients according to demographic characteristics, smoking habit, drinking habit and family history of cancer (n = 256).

Regarding cancer treatment, 180 patients (70.3%) underwent surgery, 182 (71.1%) underwent radiotherapy and 162 (63.3%) underwent chemotherapy. The most frequent tumor location was the head and neck region (57.4%; n = 147), followed by the breast (12.1%; n = 31). The most frequent histological type was squamous cell carcinoma (55.1%; n = 141). Among the patients with data on the stage of the disease when the diagnosis was made, 74 (28.9%) were in stage IV (Table 2).

**Table 2:**
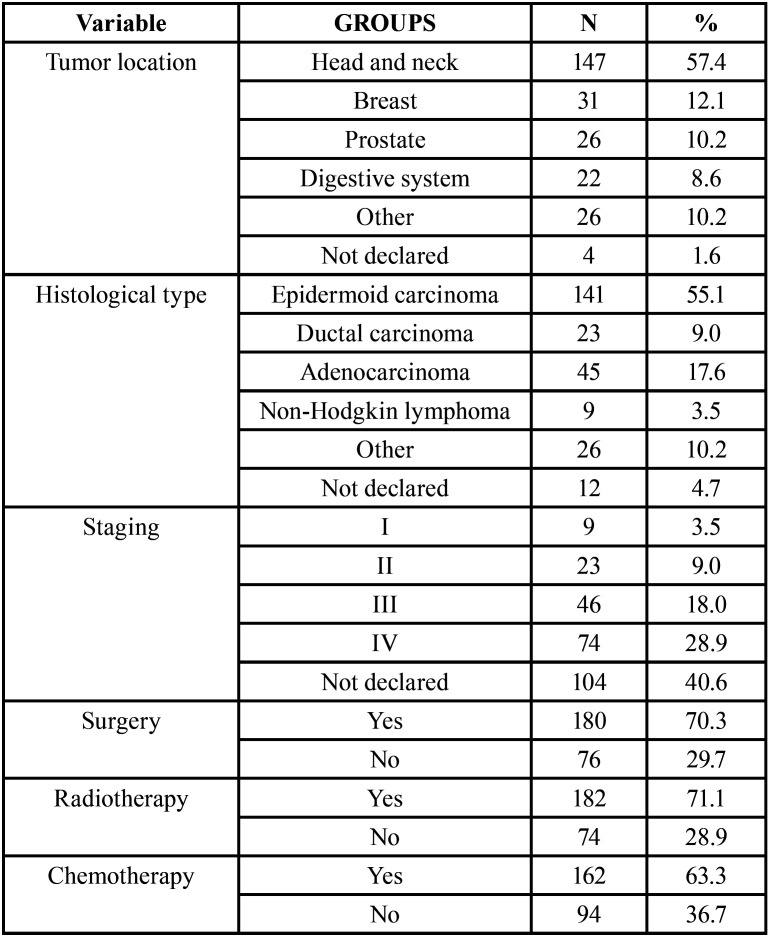
Distribution of patients according to tumor characteristics (location, histological type, staging) and cancer treatments performed (surgery, radiotherapy and chemotherapy) (n = 256).

**Figure 1:**
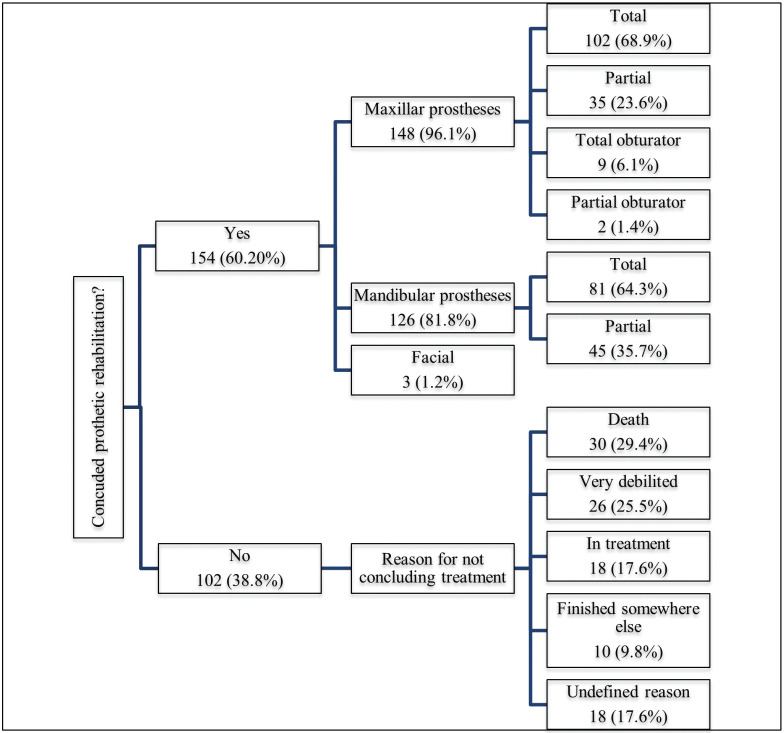
Flowchart of prosthetic rehabilitation characteristics and reasons for not completing prosthetic treatment.

Among the 256 medical records analyzed, 154 patients (60.2%) completed prosthetic rehabilitation and 102 (38.8%) did not complete rehabilitation at the Dentistry Department of the Mato Grosso Cancer Hospital. Among the 154 patients who completed the rehabilitation treatment, 148 (96.01%) received a maxillary prosthesis, among which 102 (68.9%) were total prostheses, 35 (23.6%) were partial prostheses, nine (6.1%) were total obturators and two (1.4%) were partial obturators. Among the 126 (81.8%) patients who received a mandibular prosthesis, 81 (64.3%) were total prostheses and 45 (35.7%) partial prostheses. Three patients received facial prostheses (1.2%). Among the 102 patients who did not complete rehabilitation, 30 (29.4%) died before completing prosthetic treatment, 26 (25.5%) were very debilitated by the disease or had health problems that prevented the continuation of rehabilitation, 18 (17.6%) were still undergoing rehabilitation, 10 (9.8%) finished treatment elsewhere and 18 (17.6%) qualified for “undefined reason for not concluding treatment”, as contact via telephone was not possible (Fig. 1).

Multivariate logistic regression was performed to investigate associations between the patient characteristics (age, staging, place of residence and marital status) and the completion/non-completion of prosthetic rehabilitation. Patients with advanced stages of cancer were less likely to complete treatment (*p* = 0.048), whereas no other independent variables (age, place of residence or marital status) were significantly associated with the completion/non-completion of prosthetic rehabilitation (Table 3).

**Table 3:**
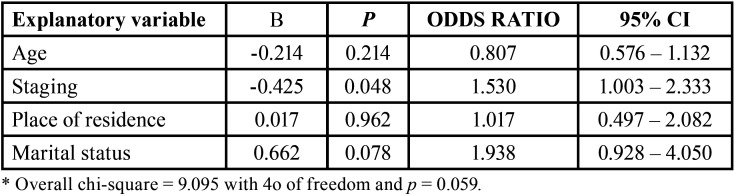
Results of multivariate logistic regression analysis of completion of prosthetic rehabilitation according to patient characteristics (age, marital status, place of residence and cancer staging).

Multivariate logistic regression was also performed to investigate associations between the patient characteristics (age, place of residence and stage of cancer) and the reason why prosthetic rehabilitation was not completed (patient was very weak, patient still undergoing prosthetic treatment, completed rehabilitation elsewhere, death or no defined reason). A statistically significant association was found between the stage of cancer and not having completed prosthetic rehabilitation due to weakness (*p* = 0.041). A significant association was also found between cancer staging and completed treatment elsewhere (*p* = 0.007), as patients in more advanced stages were less likely to complete prosthetic rehabilitation at the Cancer Hospital of Mato Grosso. Place of residence and age were not significantly associated with the reason for the non-completion of prosthetic treatment (Table 4).

**Table 4:**
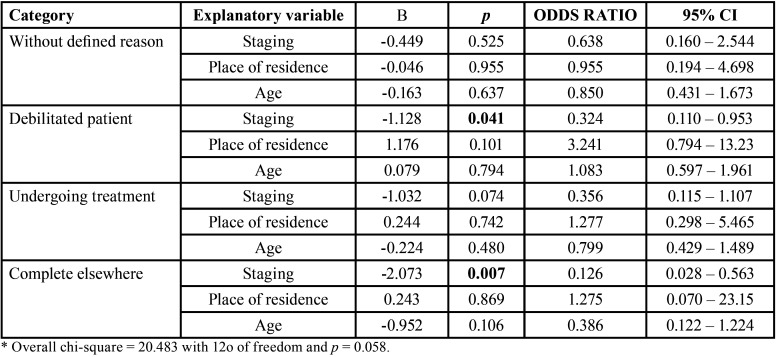
Results of multivariate ana logistic regression analysis of reasons for not completing prosthetic rehabilitation according to patient characteristics (age, place of residence and cancer staging).

## Discussion

The present findings show that cancer patients rehabilitated with dental and/or maxillofacial prostheses are mainly older adults and the main reason for not concluding treatment was the patient’s death. The population studied was predominantly male, married and retired. A total of 52% of the patients resided in municipalities other than the capital city and 70.3% had brown skin color. Caetano *et al*. (12) evaluated quality of life, body image and self-esteem in patients with sequelae following treatment for head and neck cancer, who were candidates for prosthetic rehabilitation. In the sample of 10 patients, the male sex predominated (60%), 50% were married, 30% were 51 to 60 years of age, 40% were farmers, 30% were retired and 60% resided in municipalities other than the state capital.

In a study by Rettig and D’Souza (13), the two of the main causes of head and neck cancer were the use of tobacco and alcohol. Gomes *et al*. (14) analyzed 33 patients, 84.38% and 87.50% of whom formerly used or still used tobacco and alcohol, respectively.

The anatomical sites most affected by tumors in the present sample were the head/neck (57.4%), breast (12.1%) and prostate (10.3%). Breast and prostate cancer are among the most prevalent forms of cancer in Brazil (15). The greater number of patients with tumors in the head and neck region in the present study may be explained by the fact that these patients have their oral health analyzed in the Dentistry Department before starting antineoplastic treatment and therefore already established a connection with the department, subsequently returning for oral rehabilitation.

The most frequent histopathological diagnosis was epidermoid carcinoma (55.1%; n = 141) and the most frequent disease stage was IV (28.9%; n = 74). Epidermoid carcinoma is the most frequent malignancy among tumors of the head and neck region and the sixth most common cancer globally (16).

One hundred fifty-four patients (60.2% of the population studied) completed prosthetic rehabilitation, involving a total of three facial prostheses, 148 maxillary prostheses including 11 obturators and 126 mandibular prostheses. Quispe *et al*. (17) evaluated 75 individuals, but only 30 were cancer patients. The authors assessed the need for maxillary and mandibular prostheses and found the following results: 21 patients needed a maxillary prosthesis to replace one tooth (10%) or to replace more than one tooth (33.3%), needed a combination of prostheses (13.3%) or needed a total prosthesis (13.7%) and 29 patients used a mandibular prosthesis to replace more than one tooth (70%), required a combination of prostheses (3.3%) or required a total prosthesis (23.7%). According to Joo *et al*. (7), patients undergoing oncological treatment may have several sequelae that can impair chewing function, swallowing and esthetics. Thus, the use of a total or partial obturator is an option to remedy such sequelae and enable a better quality of life. Parameswari *et al*. (18) also concluded that prosthetic rehabilitation with an obturator restores the missing intraoral structures and acts as an anatomical barrier between the oral and nasal cavities, restoring function and esthetics.

This study was conducted at the Cancer Hospital of Mato Grosso located in the city of Cuiabá, Brazil. The hospital uses a single medical record for each patient, regardless of the treatments performed in different departments of the institution. The medical records lacked some relevant information, which likely exerted an influence on the analysis and constitutes a limitation of the present study. Such a limitation is often found in studies involving the analysis of secondary data from databases not collected specifically for the research. However, this aspect is compensated by the possibility of providing information on a large number of patients over an extended period of time in a fast, agile manner (19).

## Conclusions

The patients rehabilitated with dental and maxillofacial prostheses in the present study were mainly male, older, married, individuals with brown skin residing in municipalities other than the capital city who smoked, consumed alcohol and had a history of cancer in the family. The tumor was located in the head and neck region in 57.4%. The most frequent histological type was epidermoid carcinoma (55.1%). A total of 28.9% of cases were in disease stage IV. The patients underwent surgery, radiotherapy and chemotherapy. Total prosthesis was the most common form of rehabilitation. Patients who started treatment in more advanced stages of cancer had a greater chance of not completing prosthetic rehabilitation and the main reasons for the non-completion of treatment were death and weakness.
